# Fibrothecoma of broad ligament with minor sex cord elements: Case report and brief literature review

**DOI:** 10.1097/MD.0000000000033127

**Published:** 2023-03-03

**Authors:** Yanjun Chen, Peng Zhou, Jianlin Chen

**Affiliations:** a Department of Reproductive Medicine, Longgang District Central Hospital of Shenzhen, Guangzhou University of Chinese Medicine, Shenzhen, P.R. China; b Department of Obstetrics and Gynecology, The Second Xiangya Hospital of Central South University, Changsha, P.R. China; c Department of Pathology, The Second Xiangya Hospital of Central South University, Changsha, P.R. China.

**Keywords:** broad ligament, extraovarian sex cord-stromal tumors, fibrothecoma, thecoma

## Abstract

Sex cord-stromal tumors are always found in ovary, but the occurrence of this kind of tumor at extraovarian locations is extremely rare. Up to now, the case concerning fibrothecoma of broad ligament with minor sex cord elements has not been reported, and it is extremely challenging to diagnose before surgery. In this case report, we summarized pathogenesis, clinical features, laboratory finding, imaging studies, pathology, and therapeutic schedule of this tumor, with the aim of raising awareness and attention to this type of disease.

**Patient concerns:**

A 45-year-old Chinese woman was referred to our department with intermittent lower abdominal pain for about 6 years. On examination, both ultrasonography and computed tomography revealed she had a right adnexal mass.

**Diagnosis:**

Based on the results of histology and immunohistochemistry, the final diagnosis was confirmed as fibrothecoma of broad ligament with minor sex cord elements.

**Interventions:**

This patient underwent laparoscopic unilateral salpingo-oophorectomy with excision of the neoplasm.

**Outcomes:**

Eleven days post-treatment, the patient complained that the symptoms of abdominal pain was disappeared. There is no evidence of disease recurrence 5 years after laparoscopic surgery according to the consequences of radiologic examination

**Conclusion:**

The natural history of this kind of tumor is uncertain. Although main treatment of this neoplasm might be surgical resection and good prognosis can be achieved, we believe that long-time follow-up is extremely important in all patients diagnosed as fibrothecoma of broad ligament with minor sex cord. Laparoscopic unilateral salpingo-oophorectomy with excision of the tumor should be recommended to these patients.

## 1. Introduction

Ovarian sex cord-stromal tumors, which can present as various morphology and clinical behavior, account for 7% of all ovarian tumors.^[1–3]^ These tumors are thought to originate from sex cords or stromal cells, and the former consists of granulosa cells and Sertoli cells, but the latter comprises theca cells, Leydig cells and fibroblasts.^[4,5]^ The cell types mentioned above can separately form a tumor or together with another. The morphology of fibromas and thecomas is similar, which give rise to the use of the jargon fibrothecoma. This type of tumor represents approximately 3% to 4% of all ovarian lesions.^[6]^ Another subgroup is defined as stromal tumor with minor sex cord elements, which is made up of fibrothecomatous and/or fibroma with minor sex cord elements (<10%).^[7]^ However, sex cord-stromal tumors are exceedingly rare in an extraovarian location. To the best of our knowledge, only 4 cases of stromal tumors in the broad ligament (3 cases are thecoma and 1 case is fibrothecoma) have been documented,^[8–10]^ but no case of fibrothecoma of broad ligament with minor sex cord elements has been reported in the published literature. Here, we will discuss this kind of tumor and review the literature.

## 2. Case report

A 45-year-old Chinese woman with unremarkable past medical history, gravida 4 para 2, was admitted to our department with intermittent lower abdominal pain for about 6 years and recent (2 months) progressive deterioration. Visual inspection showed that cervix was slightly enlarged accompanied with erosion. During bimanual examination, a 3 cm diameter, solid and non-mobile tumor could be palpated at the right lower quadrant. Peripheral blood cell counts showed hemoglobin was 97 g/L, however, white cell count and platelet cell count was normal, as tumor makers, including carbohydrate antigen 125, carcinoembryonic antigen, and α-fetoprotein, were within normal range.

**Figure 1.**
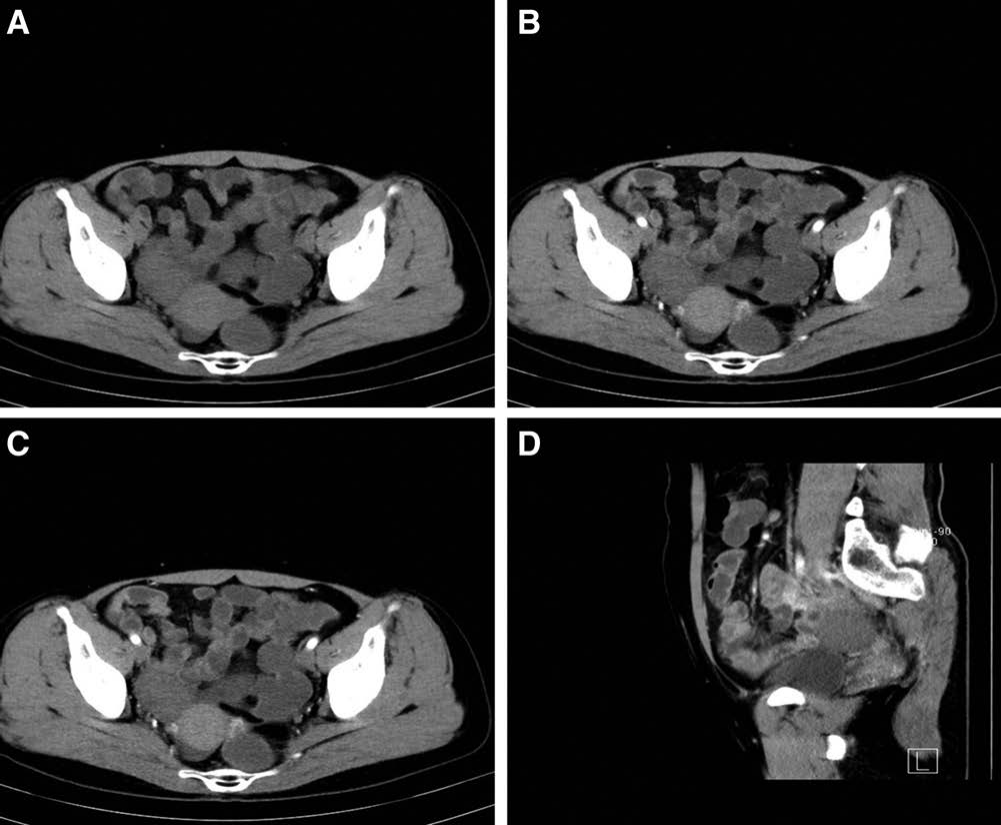
CT images of the mass. Plain CT scan showed a low density tumor with well-demarcated margin in the broad ligament (A). The neoplasm was mildly enhanced in both arterial phase (B) and venous phase (C). Three-dimensional reconstruction of the lesion on CT (D). CT = computed tomography.

Ultrasound imaging demonstrated there was a round mass (about 37 × 36 mm) in the right adnexa and the lesion was avascular. The plain computed tomography (CT) scan also revealed a low density neoplasm with well-demarcated margin, sized 3.7 × 3.0 cm, in the same location, suggestive a broad ligament leiomyoma (Fig. 1A). Contrast-enhanced CT showed the tumor was slightly in homogeneously enhanced on the arterial phase (Fig. 1B). The mass was also weakly enhanced on venous phase (Fig. 1C) and 3-dimensional reconstruction of the tumor on CT was performed (Fig. 1D). Because the malignancy of the tumor was unclear and the patient suffered chronic abdominal pain, the laparoscopic unilateral salpingo-oophorectomy was performed in our hospital.

**Figure 2.**
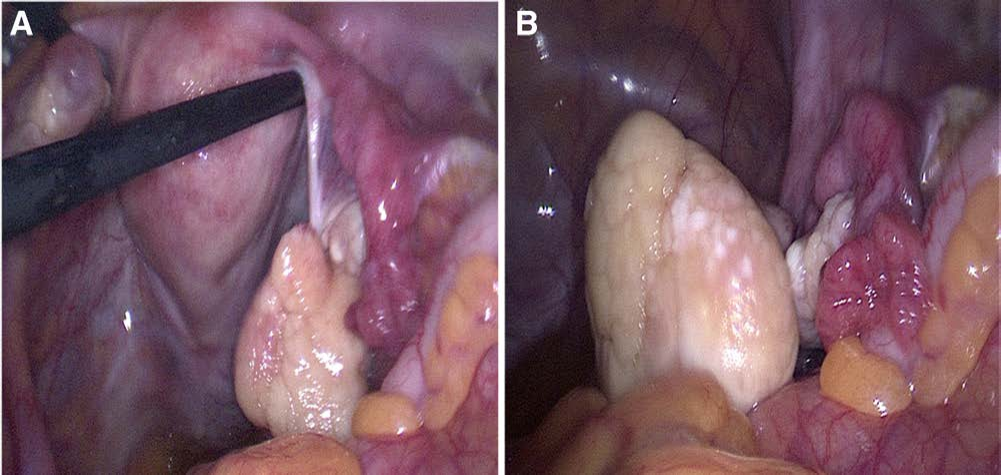
The pictures captured during the operation. Figures (A) and (B) presented the surface appearance of the tumor.

During intraoperative period, local adhesions could be observed between the right adnexa and the bowel and mesentery. After carefully separating the adhesions step by step, we observed that there was a tumor, sized 4 × 4 × 3 cm, with a clear margin, on the right broad ligament, but the fallopian tubes, ovaries and uterus were normal and not invaded (Fig. 2A). The external surface of the neoplasm was white to gray, and the tumor was firm, smooth and lobulated with no hemorrhage and necrosis (Fig. 2B). The amount of bleeding during the operation was about 20 mL.

**Figure 3.**
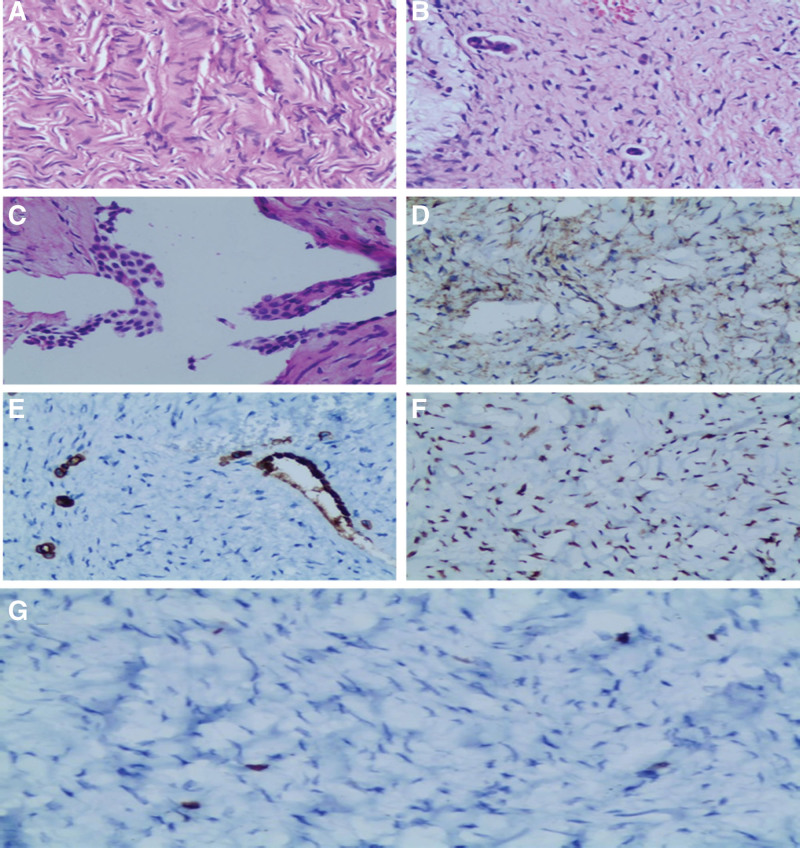
Histological and immunohistochemical images of the mass. Histopathology showed that the tumor was a mesenchymal neoplasm (A), including very few tubuliform structures (B). Sacculiform structures of the mass were covered with granulosa cells (C). Immunohistochemistry revealed positive staining with inhibin (D), CK (E), and WT1 (F). Ki-67 staining was positive in 2% of tumor cells (G) (magnification 200).

The resected specimen consisted of the lesion and the right adnexa, and the tumor had no capsule. Microscopic examination showed that it was a mesenchymal neoplasm mainly made up of epithelioid cells (Fig. 3A), which contained very few tubuliform structures (Fig. 3B). The tumor showed that the sacculiform structures were covered with granulosa cells (Fig. 3C). Immunohistochemistry revealed positive staining with inhibin (Fig. 3D), CK (positive expression on tubuliform structures) (Fig. 3E), and WT1 (Fig. 3F), as the Ki-67 proliferative index that belongs to these tumor cells was about 2% (Fig. 3G). Cellular atypia and nuclear atypia were not conspicuous, as the nuclear grooves were inapparent. Immunohistochemistry also showed positive staining with β-catenin, Vim, and CD99. There was focal staining with smooth muscle actin and calretinin, but the S100, CD34, Dog-1, HCAL, HHF35, and P53 immunohistochemistry outcomes were negative (data not shown).

The diagnosis of fibrothecoma of broad ligament with minor sex cord elements was made in our patient on the grounds of expression of several markers that are the characteristics of ovarian sex cord-stromal tumors and ruling out of other neoplasms.

Six days after the operation, the patient complained that the symptoms of abdominal pain was relieved. Eleven days post-treatment, the abdominal pain was disappeared. After she was discharged from the hospital, she had ultrasonography or CT examination every 3 months. There is no evidence of disease recurrence 5 years after surgery.

## 3. Discussion

Primary tumors that occur in broad ligament are extremely rare, which include leiomyoma, cyst, neurilemmoma, teratoma, lipoma and hemangioma and so on, but before now, only 3 cases of stromal tumors in the broad ligament have been documented in English literature,^[8,9]^ and another case was reported in Italian.^[10]^ Two were classified as fibrothecoma,^[8]^ two as thecoma,^[9,10]^ but our case was diagnosed as fibrothecoma of broad ligament with minor sex cord elements. There was a large mass in 1 patient, and the symptoms in the other 3 patients were not clear due to the unavailable literature (one of the case was presented in Italian,^[10]^ so the content of this paper was excluded from our discussion section), but our patient had a chief complaint of abdominal pain. The sizes were different between these tumors, and the diameter of one of the case was 20 cm,^[9]^ which is the 5 times the diameter of our case. Patients with no obvious symptoms or presenting as slight symptom may be the reasons why the neoplasms could be large in size.

The broad ligament consisting of 2 segments, one of which is mainly made up of fibroconnective tissue, and another segment contains a large number of vessels, muscles and connective tissue. Extraovarian sex cord-stromal tumors, according to the related references, are exceedingly rare. Meanwhile, different anatomical sites of this kind of tumor have also been reported, which include fallopian tube, mesovarium, retroperitoneum, adrenal gland, mesentery, broad ligament and an umbilical hernia sac.^[11]^ However, if a lesion is considered as primary broad ligament tumor, the most important thing is that the ipsilateral ovary should appear normal, with wholly intact cortex. The histogenesis of extraovarian sex cord-stromal tumors is still uncertain, but ectopic gonadal stromal tissue is thought to be one of the histogenetic origin of these tumors.^[12,13]^ Up to now, pelvis and retroperitoneum are the most common location of the origin of extraovarian sex cord-stromal tumors.^[11]^

A review of the cases showed that laboratory results of serological tumor markers, which include carcinoembryonic antigen, α-fetoprotein and carbohydrate antigen 125, were normal. Two of the cases (fibrothecoma) had no function of hormone production, and 1 case (thecoma) was found to produce estradiol and estrone.^[8,9]^ Before operation, the examination of testosterone and estradiol levels had not been performed in our patient, but both of them were within the normal range when they were tested postoperatively, which indicated that our case did not generate hormone.

For sex cord-stromal tumors, it is difficult to differentiate a subtype only in clinical manifestations and laboratory examination, so the imaging findings should be performed to provide valuable diagnostic information. Ultrasound is the most common methods for this type of tumor. According to the literature, ultrasonography was implemented in one of the cases of stromal tumors in the broad ligament. Ultrasonography revealed a big mass and ascites, and most of the neoplasm was solid and partly cystic. In our case, the lesion appears solid and avascular, with a heterogeneous ultrasonic echo. To get a better understanding of the disease, we believe that the enhanced ultrasonography should be recommended. Reviewing the literature,^[14]^ if a lesion without calcification could cast a dense acoustic shadow in ultrasonography, it could be regarded as a rather specific finding of the ovarian thecoma. CT might be the optimal diagnostic option for the diseases, and CT was carried out in one of the case according to the literature. Generally, CT appearances of pure thecomas or thecomas with scarce fibrotic components of ovary are not distinct, and precontrast CT of our patient revealed a low density neoplasm with well-demarcated margin, as the lesion was slightly enhanced on the arterial phase as well as the venous phase. A research by Zhang et al^[15]^ prospectively estimated the magnetic resonance imaging (MRI) characteristics of 18 cases of thecomas/fibrothecomas of ovary. The authors drawn a conclusion that 61.1% of thecomas/fibrothecomas were homogenous tumors and appeared isointense to the myometrium on diffusion-weighted imaging MRI. Pure thecomas of ovary tend to demonstrate greater hyperintensity on T2-weighted images. Due to the vascularization of theca cells, a more apparent contrast-enhancement could be seen in thecomas/fibrothecomas. We also think that MRI might exert an pivotal role in the diagnosis of this disease.

Upon gross examination, ovarian sex cord-stromal tumors are mostly solid masses and the cut surface of them shows grayish white and yellowish areas.^[6]^ But for extraovarian sex cord-stromal tumors, 1 case (thecoma of broad ligament) showed that the lesion presented as solid nodular parts with brownish-yellow appearance, and yellowish clear fluid was seen in several cystic spaces of the neoplasm. The surface of the neoplasm was white to gray in our case. Pathologically, according to the literature,^[16]^ ovarian sex cord-stromal tumors are made up of uniform spindle cells that can arrange in sheets, and the amount of eosinophilic cytoplasm was scant to moderate, with round nuclei but without nuclear atypia or nuclear grooves. Theca cells that usually surround the ovarian follicles compose ovarian thecomas, which demonstrate estrogenic activity in most cases.^[17,18]^ On immunohistochemistry, ovarian thecomas could show positivity for vimentin, WT1 and inhibin in. Interestingly, immunohistochemistry in our case reveals positive staining with WT1, which is rarely expressed in sex cord-stromal tumors of broad ligament. Minor sex cord element elements (granulosa cells) have also been seen in our patient.

Differential diagnoses of the tumor that we report may depend on morphology of the neoplasm and still on the age of different patients. The characteristics of kinds of differential diagnoses are too more to be discussed detailed, and the differential diagnoses probably include epithelioid smooth muscle tumors, malignant melanoma, metastatic carcinoma from other places, a variety of sarcomas, intra-abdominal desmoplastic small round cell tumor, multifarious small round blue cell tumors, malignant mesothelioma, malignant gastrointestinal stromal tumors, small cell carcinoma of the ovary of hypercalcemic type (this is easily to be ruled out if there is no mass on the ovary), and granulosa cell tumors.^[19]^

Given all factors above, it is challenging for gynecologist to make a correct diagnosis on this kind of tumor before surgery or without a biopsy. Thecomas represent approximately 0.5% to 1% of all primary ovarian tumors.^[15,20]^ At the same time, these tumors are more likely to be found in postmenopausal women and most cases are regarded as benign neoplasms.^[20]^ Although only several cases of thecoma/fibrothecoma of broad ligament had been reported, uncertain malignant potential of these neoplasms could not be ignored. Surgery was performed in all of the 3 cases, and in our opinion, surgical resection may be the gold standard for the treatment of these tumors. For extraovarian sex cord-stromal tumors, fertility-sparing surgery, such as preservation of the ipsilateral ovary, is a significant consideration in young patients who desire future childbearing.

## 4. Conclusion

Extraovarian sex cord-stromal tumors are rarely documented, we are the first one to report fibrothecoma of broad ligament with minor sex cord elements in the world. No specific clinical symptoms could be observed before surgery. Thus, it is difficult for doctors to make a correct preoperative diagnosis. According to the literature, we believe that CT, MRI and enhanced ultrasonography should be recommended to this type of tumor, especially MRI. Additionally, the final diagnosis must depend on the immunohistochemical staining results. Owing to the semi malignant potential of these tumors, surgery should be performed, and laparoscopic unilateral salpingo-oophorectomy with excision of the neoplasm should be recommended to the postmenopausal women. After operation, long-time follow-up is extremely important in all patients with sex cord-stromal tumors of broad ligament.

## Acknowledgments

Pathology department of the second Xiangya Hospital provided the Histological and immunohistochemical figures for the authors.

## Author contributions

**Conceptualization:** Jianlin Chen.

**Data curation:** Peng Zhou, Jianlin Chen.

**Investigation:** Peng Zhou.

**Project administration:** Yanjun Chen.

**Software:** Yanjun Chen.

**Writing – original draft:** Yanjun Chen, Jianlin Chen.

**Writing – review & editing:** Yanjun Chen, Jianlin Chen.
